# Supplementary material for: Whose centre is it anyway? Defining person-centred care in nursing: An integrative review
Source: PLoS One. 2020 Mar 10;15(3):e0229923. doi: 10.1371/journal.pone.0229923 (PMC7064187; doi:10.1371/journal.pone.0229923)
Supplement: Attachment — Submitted filename: PONE-D-19-26103_review.docx [file pone.0229923.s002.docx]

This is an interesting paper that has the potential to inform policy change in the future. The review itself seems quite strong, however, the international/Australian focus needs more clarity. In the literature search strategy you have chosen to include multiple jurisdictions which have important differences in healthcare system functioning. However, in each section when you give examples they are only focused on the Australian context. While I understand that for the definition of PCC the inclusion of more broad context is helpful, consider carefully how the operationalization will be different in different jurisdictions and the policy/practice challenges. I suggest you either make it clear that this global review of definitions is only being applied to the Australian context, or diversify your examples throughout. This will also be important for the corresponding terminology that you use – the term consumer for example is rarely used in the Canadian healthcare context so it will be important early in the paper to define such terms and outline which contexts they are used and comparable terms in each of the other jurisdictions. Also related to terminology in Canada, we would refer only to the ‘healthcare system’, not the ‘health system’ as there is an acknowledgement that it is not about preventative health.

Here are some more minor changes throughout:

**Introduction:**

Page 3, line 47-49 “The concept of Person-Centred Care (PCC) is used to describe the role of the patient within the health system and the way in which care is provided to the patient [2,3].”

- This is to describe a certain model for the role of the patient – not the role of patients in general – this implies it is a way to describe patients in all care models

Page 3, line 52-58 – accreditation is based on jurisdiction – which jurisdiction are you referring to? Same with nursing professional standards.

Page 4, line 59 – healthcare not health

Page 4, line 63 – is this different nursing contexts as in hospitals vs community or different countries where they practice?

**Background:**

Page 5, line 84 – when was this report created? What happened between 1977 and 2000? Add a sentence here, likely wasn’t a 20-30 year period without any changes.

Page 5, line 86 – is this the first definition of PCC? It’s the first you mention as far as I can tell for this specific term, might be important to mention this if it is the first attempt to define it.

Line 89 – is this WHO framework? Unclear who’s framework – please specify

Line 94 – awkward “of their own care” consider changing to about their own care or over their own care.

Line 96-97 – this quote is cut a little awkwardly – consider adding a word in square brackets to make it flow better like [involving].

Line 98 – from not form

Again your aim and scope doesn’t specify an Australian context, rather an international application. Either make clear in the introduction that this paragraph about Australia’s ACSQHC is an example from the various jurisdictions. Potentially consider including an example from another country to support the international focus of the paper.

Paragraph starting line 104 seems disconnected from previous paragraph. Consider adding a sentence at the beginning that bridges and waiting until the second sentence to list examples of frameworks. Consider moving the sentence on 110 to this paragraph as it justifies the move from PCC in general to nursing frameworks specifically.

Line 114-116 – combine these 2 short sentences.

**Aim**:

- The first aim – to better understand the literature – seems vague, can you be more specific here? Consider combining with the search question – ie the aim could be to understand better from the literature how nurses operationalize the definition.

**Methods**:

Line 144 – you don’t specify you use this framework, make this clear

Figure 1 – are you missing the arrow from screening to eligibility?

How many papers are citing the same papers?

**Findings**:

Consider creating a figure or table summarizing each of the themes and subthemes to allow a visualization of your results that is easier for readers to refer to.

Line 200 – ‘based’ not ‘base’.

Line 237 – take out the “the” before people.

Line 240 – need to define the term medical officer, either in this paragraph or as a footnote.

Line 281 – access to what?

Line 290 – if measuring PCC is important to the discussion and still part of the papers you reviewed it would be helpful to add a sentence or two here to justify why you didn’t include them.

**Discussion**:

Lines 302-305 – abrupt switch between these two sentences, consider a sentence in between moving from stating it’s a model of care to what the reader must assume is the definition that emerged from the review.

Line 325 – they are ‘an’ intrinsic element.

Line 330 – core business – consider core competency or another word that is more in line with healthcare and patient centredness.

Line 354 – this sentence seems unfinished – should it say “giving choice and decision-making power to patients”?

Line 360 – again should be healthcare system throughout.

Again this is an Australian example, consider diversifying or adding a second country example to make the argument that your paper has international scope.

**Implications for practice**:

Again this is another section to clarify the jurisdiction you are talking about and be careful about the suggestions you are making here and in the conclusion about policy and practice. It could be helpful to say that across the western countries included general things need to change and give a couple of specific examples in different countries.
