# Supplementary material for: Whose centre is it anyway? Defining person-centred care in nursing: An integrative review
Source: PLoS One. 2020 Mar 10;15(3):e0229923. doi: 10.1371/journal.pone.0229923 (PMC7064187; doi:10.1371/journal.pone.0229923)
Supplement: Attachment — Submitted filename: Response to Reviewers letter.docx [file pone.0229923.s003.docx]

PLOS One	10/01/2020

To the Editor and Reviewers.

Thank you for the comprehensive feedback on the manuscript *Whose Centre is it Anyway? Defining Person-Centred Care in Nursing; An Integrative Review.* The valuable feedback has been considered and changes have been made as per the table below. Please find attached a revised copy of the manuscript with tracked changes and a complete copy with changes embedded. Once again, we thank you for your time and consideration and we look forward to your consideration of this new version.

Yours sincerely

Amy-Louise Byrne (Corresponding author)

| No | Feedback | Action |
| --- | --- | --- |
| 1 | Ensure manuscript meets Plus Ones style requirement templates | The article meets the requirements for PLOS One, minor changes have been made to the references of Fig and headings as per the example template |
| 2 | Ensure the study type of integrative review is in the title and the full articles for the method have been cited | The article is an integrative review, and this is stated in the title. The work of Whitmore and Knafl has been used as the protocol and the search strategy is registered with Prospero ID number 148778. This is currently stated in the paper. |
| 3 | Refer to table 2 in the text | Reference to table 2 added to text |
| 4 | Make reference of figure 1 in the text | Sentences added to text which references Figure 1 |
|  | Reviewer 1 |  |
| 5 | It is not clear how quality appraisal using CASP was applied to the studies included | The article states 'To ensure adequate rigour, reliability and relevance, all articles were evaluated against the Critical Appraisal Skills Programme (CASP) systematic and qualitative review checklists [24,25] by the lead author and reviewed by a senior researcher on the team. The relevance of the papers and the quality of the reviews/articles themselves was appraised. All articles were appraised against the aims of this review.'  The CASP process is also demonstrated in the PRSIMA chart Figure 1 |
| 6 | The results are presented concisely, clearly and make sense with respect to the aim of the review but could have been longer to elaborate on the characteristics of the papers included in the review. | Table 2 provides a break-down of the type and characteristics of the included articles, including the design methods, their findings and their population. This is now referenced on page 9 'Table 2 demonstrates the characteristics of the articles reviewed including their design methods, populations and findings.' |
| 7 | There could have been a better debate highlighting the linkage or differentiating features of PCC from similar concepts such as patient centered care or patient and family centered care. | Updated background to include 'The concept of PCC continues to evolve, notably in the change to ‘person’ rather than ‘patient’ in recognition of the whole person, not simply the disease process. Other variables such as Family-centred care are used more in the context of aged care and paediatrics [16,17]. ' Family CC is also discussed on page 29 and in the summary findings table |
| 8 | The fact that authors attempted to highlight current literature on person centered care, this could have been more interesting if they could have highlighted the differences in terms of definition by the different scholars or institutions. | Table 2- in the findings and comments section provides a section on the definitions provided by the included articles. Definitions from IOM (USA), WHO and the ACSQHC (Aus) are provided |
| 9 | as this is an integrative review, it could offer more in the way of future direction for defining the concept of PCC. | Within the implications of practice section, we have added 'Any definition provided should carefully consider how PCC is measured and prioritised within the healthcare system, which has the potential to move the concept from its current rhetorical nature, to a genuine commitment and priority of nurses and services.' |
|  | Reviewer 2 |  |
| 10 | The review itself seems quite strong, however, the international/Australian focus needs more clarity. In the literature search strategy you have chosen to include multiple jurisdictions which have important differences in healthcare system functioning. However, in each section when you give examples they are only focused on the Australian context. While I understand that for the definition of PCC the inclusion of more broad context is helpful, consider carefully how the operationalization will be different in different jurisdictions and the policy/practice challenges. I suggest you either make it clear that this global review of definitions is only being applied to the Australian context, or diversify your examples throughout. | Excerpt has been added to clarify that while that search strategy is inclusive of other regions the information will be related to the Australian context; 'While the search strategy includes international literature, this will be related back to the Australian context, in order to understand how PCC operates within Australia.' |
| 11 | This will also be important for the corresponding terminology that you use – the term consumer for example is rarely used in the Canadian healthcare context so it will be important early in the paper to define such terms and outline which contexts they are used and comparable terms in each of the other jurisdictions. Also related to terminology in Canada, we would refer only to the ‘healthcare system’, not the ‘health system’ as there is an acknowledgement that it is not about preventative health. | The following excerpt was added to the bottom of the background. 'This review uses the term person rather than patient in recognition of the person as a whole. Where clarity is required, the term healthcare consumer is used; a term frequently used in Australia.  Term changed to healthcare throughout the article |
| 12 | Page 3, line 47-49 “The concept of Person-Centred Care (PCC) is used to describe the role of the patient within the health system and the way in which care is provided to the patient [2,3].” - This is to describe a certain model for the role of the patient – not the role of patients in general – this implies it is a way to describe patients in all care models | Amended to state The concept of Person-Centred Care (PCC) is used to describe a certain model for the role of the patient within the healthcare system and the way in which care is provided to the patient [2,3]. |
| 13 | Page 3, line 52-58 – accreditation is based on jurisdiction – which jurisdiction are you referring to? Same with nursing professional standards. | Clarified to state this is within the Australian Context |
| 14 | Page 4, line 59 – healthcare not health | Changed to healthcare |
| 15 | Page 4, line 63 – is this different nursing contexts as in hospitals vs community or different countries where they practice? | Changed to 'different practice settings and specialities' |
| 16 | Background- Page 5, line 84 – when was this report created? What happened between 1977 and 2000? Add a sentence here, likely wasn’t a 20-30 year period without any changes. | 2001 added to IOM reference.  A linking sentence has now been included which reads; 'Over the succeeding years, this model of care and the notion of patient centre care continued to evolve, with many iterations of the term moving with the changing climate of healthcare systems. ' |
| 17 | Page 5, line 86 – is this the first definition of PCC? It’s the first you mention as far as I can tell for this specific term, might be important to mention this if it is the first attempt to define it. | The IOM provided one of the first contemporary definitions, stating that PCC 'Encompasses…' added to text |
| 18 | Line 89 – is this WHO framework? Unclear who’s framework – please specify | Clarified to add  'The World Health Organization continues to advocate for integrated care that is in tune with the patient’s wants and needs through the framework on Integrated People-Centred Care. This includes the vision that *“all people have equal access to quality health services that are co-produced in a way that meets their life course needs”.* |
| 19 | Line 94 – awkward “of their own care” consider changing to about their own care or over their own care. | Changed to 'over their own care' |
| 20 | Line 96-97 – this quote is cut a little awkwardly – consider adding a word in square brackets to make it flow better like [involving]. | [Involving] added to quote |
| 21 | Line 98 – from not form | corrected |
| 22 | Again your aim and scope doesn’t specify an Australian context, rather an international application. Either make clear in the introduction that this paragraph about Australia’s ACSQHC is an example from the various jurisdictions. Potentially consider including an example from another country to support the international focus of the paper. | Modified method section to read; 'The international context off PCC is then specifically related to the Australian context.' |
| 23 | Paragraph starting line 104 seems disconnected from previous paragraph. Consider adding a sentence at the beginning that bridges and waiting until the second sentence to list examples of frameworks. Consider moving the sentence on 110 to this paragraph as it justifies the move from PCC in general to nursing frameworks specifically. | Bridging sentence included and sentence at 110 moved to be included in the paragraph |
| 24 | Line 114-116 – combine these 2 short sentences. | Now reads 'Consumers of healthcare navigate a complex and fragmented system, with fragmentation leading to patients feeling lost, and a decrease in the quality of services offered' |
| 25 | - The first aim – to better understand the literature – seems vague, can you be more specific here? Consider combining with the search question – ie the aim could be to understand better from the literature how nurses operationalize the definition. | Suggestion accepted- now reads 'The aim of this literature review was to understand better from the literature how nurses operationalise the definition of PCC.' |
| 26 | Line 144 – you don’t specify you use this framework, make this clear | Amended to clarify that this framework was used by the author to identify the max no of articles. |
| 27 | Figure 1 – are you missing the arrow from screening to eligibility? | Corrected |
| 28 | How many papers are citing the same papers? | The Prisma provides the duplicates removed. Table 2 provides a summary of the common authors and articles cited by each article |
| 29 | Findings- Consider creating a figure or table summarizing each of the themes and subthemes to allow a visualization of your results that is easier for readers to refer to. | Summary table created and added as Table 3 |
| 30 | Line 200 – ‘based’ not ‘base’. | Corrected |
| 31 | Line 237 – take out the “the” before people. | Corrected |
| 32 | Line 240 – need to define the term medical officer, either in this paragraph or as a footnote. | Changed to Doctors |
| 33 | Line 281 – access to what? | Access removed |
| 34 | Line 290 – if measuring PCC is important to the discussion and still part of the papers you reviewed it would be helpful to add a sentence or two here to justify why you didn’t include them. | Amended to read 'This review excluded articles related to the measurement of PCC as the primary aim was to find how PCC was defined, however this was still very much a part of the discussion around the meaning and practice of PCC.' |
| 35 | Discussion- Lines 302-305 – abrupt switch between these two sentences, consider a sentence in between moving from stating it’s a model of care to what the reader must assume is the definition that emerged from the review. | To be clear the authors have not stated that it is a Model of care- but rather the way in which nurses deliver care. This has been clarified to read ' The review demonstrates that the concept of PCC is indeed a method of providing care, or the way in which nurses deliver care. |
| 36 | Line 325 – they are ‘an’ intrinsic element. | Corrected |
| 37 | Line 330 – core business – consider core competency or another word that is more in line with healthcare and patient centredness. | Amended to competency |
| 38 | Line 354 – this sentence seems unfinished – should it say “giving choice and decision-making power to patients”? | Suggestion accepted and incorporated into the sentence. Now reads 'Kim goes on to say that these dimensions vary with individual nurses and changing clinical situations; which seems to fit with the current confusion about PCC giving choice and decision-making power to patients' |
| 39 | Line 360 – again should be healthcare system throughout. | Amended throughout |
| 40 | Again this is an Australian example, consider diversifying or adding a second country example to make the argument that your paper has international scope. | See above- amended in the methods section and in the search strategy |
| 41 | Again this is another section to clarify the jurisdiction you are talking about and be careful about the suggestions you are making here and in the conclusion about policy and practice. It could be helpful to say that across the western countries included general things need to change and give a couple of specific examples in different countries. | See above- amended in the methods section and in the search strategy |
