# Supplementary material for: Whose centre is it anyway? Defining person-centred care in nursing: An integrative review
Source: PLoS One. 2020 Mar 10;15(3):e0229923. doi: 10.1371/journal.pone.0229923 (PMC7064187; doi:10.1371/journal.pone.0229923)
Supplement: Attachment — Submitted filename: Response to Reviewers letter 2.docx [file pone.0229923.s004.docx]

PLOS One	05/02/2020

To the Editor and Reviewers.

Thank you for the feedback on the manuscript *Whose Centre is it Anyway? Defining Person-Centred Care in Nursing; An Integrative Review.* The valuable feedback has been considered however no changes have been made as per the response in the table below. Once again, we thank you for your time and consideration and we look forward to your consideration of this version.

Yours sincerely

Amy-Louise Byrne (Corresponding author)

| **Feedback** | **Response** |
| --- | --- |
| The authors have sufficiently responded to the previous comments and thanks for submitting the revision. However, they could consider expanding on the background by including more literature from other countries which have significantly contributed to literature person-centered care. Some suggestions, there is plenty of literature coming from the University of Gothenburg Center or Person centers care GPCC and Finland on this topic. This will reflect a global picture on the topic. Were there any assumptions that the authors made about including the countries specified? | Thank you for your feedback and the suggested changes. The authors have articled in the search strategy that the paper includes articles related to specific countries; ‘Publications from Australia and New Zealand, Canada, USA, Europe, UK and Ireland were included to gain an understanding of PCC in the western context.’ The background information is also inclusive of this context and we feel that the suggested work from the GPCC is not relate to the aim of this paper (defining person-centred care) and hence does not add value to the background.  The articles included in this integrative review include many international articles including three from Scandinavian countries (Arakelian et al., Edvardsson et al., Bala et al).  We have investigated the literature from the GPCC and agree that there are some exciting developments currently underway around PCC, particularly the ‘Mapping Person-centred care on European capital cities’ and ‘Translating person-centred care from theory to practice’. We will consider this future research through the development of our project. |
